# A P_3A_-Type ATPase and an R2R3-MYB Transcription Factor Are Involved in Vacuolar Acidification and Flower Coloration in Soybean

**DOI:** 10.3389/fpls.2020.580085

**Published:** 2020-11-30

**Authors:** Jagadeesh Sundaramoorthy, Gyu Tae Park, Jeong-Dong Lee, Jeong Hoe Kim, Hak Soo Seo, Jong Tae Song

**Affiliations:** ^1^School of Applied Biosciences, Kyungpook National University, Daegu, Korea; ^2^Department of Biology, Kyungpook National University, Daegu, Korea; ^3^Department of Plant Bioscience, Seoul National University, Seoul, Korea

**Keywords:** flower color, vacuolar acidification, H^+^ P-ATPase, GmPH5, GmPH4, soybean (*Glycine max*)

## Abstract

The determination of flower color mainly depends on the anthocyanin biosynthesis pathway and vacuolar pH; however, unlike the former, the mechanism of vacuolar acidification in soybean remains uncharacterized at the molecular level. To investigate this mechanism, we isolated four recessive purple–blue EMS-induced flower mutants from the purple flower soybean cultivar, Pungsannamul. The petals of all the mutants had increased pH compared with those of wild Pungsannamul. One of the mutants had a single nucleotide substitution in *GmPH4*, a regulator gene encoding an MYB transcription factor, and the substitution resulted in a premature stop codon in its first exon. The other three mutants had nucleotide substitutions in *GmPH5*, a single new gene that we identified by physical mapping. It corresponds to *Glyma.03G262600* in chromosome 3 and encodes a proton pump that belongs to the P_3A_-ATPase family. The substitutions resulted in a premature stop codon, which may be a defect in the ATP-binding capacity of GmPH5 and possibly a catalytic inefficiency of GmPH5. The result is consistent with their genetic recessiveness as well as the high pH of mutant petals, suggesting that GmPH5 is directly involved in vacuolar acidification. We also found that the expression of *GmPH5* and several putative “acidifying” genes in the *gmph4* mutant was remarkably reduced, indicating that GmPH4 may regulate the genes involved in determining the vacuolar pH of soybean petals.

## Introduction

Flower color is manifested by three main classes of pigments: anthocyanins, carotenoids, and betalains (Brockington et al., 2015). Anthocyanins and carotenoids can be abundantly found in angiosperms, whereas betalains are only found in Caryophyllales (Grotewold, 2006). Carotenoids are hydrophobic compounds that are produced and stored in plastids (Ng and Smith, 2016), and they are responsible for the yellow and orange colors in ornamentals such as marigold, daffodil, *Lilium*, and *Rosa* (Grotewold, 2006). In contrast, anthocyanins are water-soluble flavonoids stored in vacuoles; they contribute to various flower colors, such as red, pink, blue, and purple (Tanaka et al., 2008; Ng and Smith, 2016). Of these pigments, anthocyanins are the most extensively studied owing to their wide distribution among angiosperms (Grotewold, 2006). Furthermore, anthocyanins are the predominant pigments in ornamentals such as petunia, *Ipomoea*, and snapdragon as well as soybean (Grotewold, 2006; Iwashina et al., 2008).

The early steps in the anthocyanin biosynthesis pathway are catalyzed by chalcone synthase, chalcone isomerase, and flavanone 3-hydroxylase, whereas the later steps are mediated by dihydroflavonol-4-reductase, anthocyanin synthase, and flavonoid 3-*O*-glucosyltransferase (3GT) (Morita and Hoshino, 2018). Anthocyanidin 3-glucosides are the first stable anthocyanins synthesized by 3GT-catalyzed glycosylation at the third carbon position of anthocyanidin aglycones (Morita and Hoshino, 2018). In most higher plants, the anthocyanin biosynthesis pathway is reportedly regulated by a MBW complex comprising transcription factors that contain MYB, basic-helix-loop-helix (bHLH), and WD-repeat (WD40) domains (Hartmann et al., 2005; Lepiniec et al., 2006; Quattrocchio et al., 2006a; Solfanelli et al., 2006; Gonzalez et al., 2008; Albert et al., 2014).

Plant MYB proteins contain a single or multiple repeat of structurally conserved MYB DNA-binding domain(s). Among the MYB protein families, the largest is the two-repeat class (R2R3), which is associated with the anthocyanin pathway (Allan et al., 2008). In several plant species, such as petunia, *Phalaenopsis* orchids, *Antirrhinum*, and soybean, the activity of MYB proteins influences differential pigmentation in different parts of the petal (Quattrocchio et al., 1998; Schwinn et al., 2006; Takahashi et al., 2013; Hsu et al., 2015). R2R3-MYB transcription factors of the MBW complex are considered as a key determinant controlling distinct pigmentation patterns throughout the plant, whereas WD40 and bHLH transcription factors are shared between floral and vegetative pigmentation regulation (Albert et al., 2011). In petunia, ANTHOCYANIN2 (AN2) and AN4 encode members of the R2R3-MYB transcription factor family that regulates anthocyanin synthesis in floral tissues (Quattrocchio et al., 1999; Albert et al., 2011), whereas PH4 is another MYB transcription factor that regulates vacuolar acidification (Quattrocchio et al., 2006b).

Vacuolar pH plays an important role in hueing anthocyanin pigments, providing varying degrees of flower color. In all cells, the vacuolar lumen has a lower pH than the surrounding cytoplasm. In petunia, the hyperacidity of the vacuoles of flower petals results in red-colored flowers (Faraco et al., 2014). Mutations affecting vacuolar pH regulation lead to bluish flower color and increased pH of petal homogenates (Verweij et al., 2008; Faraco et al., 2014). In most plant cells, the pH gradient across the tonoplast is generated by vacuolar ATPases (V-ATPase) or H^+^-pyrophosphatases (Eisenach et al., 2014). However, recent studies involving flower color mutants of petunia and other ornamental plants revealed that phosphorylated ATPases (P-ATPases) also reside in the tonoplast and play a key role in determining the vacuolar pH of petals (Verweij et al., 2008; Faraco et al., 2014).

Petunia has seven distinct loci (*PH1* to *PH7*) controlling flower coloration. Wild-type (WT) petunia petals with accumulated cyanidins display red color, and vacuolar pH is ~5.5 when all *PH* genes are functional (Faraco et al., 2014). However, mutations in any of the seven loci lead to an increase in the vacuolar pH of petals, up to ~6.0, thereby exhibiting blue color (Spelt et al., 2002; Faraco et al., 2014). In petunia, the activity of the MBW complex is sometimes enhanced by another transcription factor that contains the WRKY domain (Verweij et al., 2016). The genes involved in vacuolar acidification are activated by MBWW complex proteins, including PH4 (an MYB protein), AN1 (a bHLH protein), AN11 (a WD40 protein), and PH3 (a WRKY protein) (Spelt et al., 2002; Koes et al., 2005; Quattrocchio et al., 2006b). Moreover, *PH5* and *PH1* are the most important downstream structural genes involved in vacuolar acidification (Verweij et al., 2008; Faraco et al., 2014). PH5 is a P_3A_-ATPase-type proton pump, whereas PH1 is a P_3B_-ATPase-like bacterial Mg^2+^ transporter, and these P-type ATPases are located in the tonoplast (Verweij et al., 2016). Although PH1 has no H^+^ transport activity on its own, it can physically interact with PH5, thereby promoting the proton-pumping activity of PH5 (Li et al., 2016).

In recent years, flower color variations in soybean (*Glycine max*) have been extensively studied. To date, six genes have been identified: five structural genes (*W1, W3, W4, Wm*, and *Wp*) encoding enzymes involved in flavonoid biosynthesis and one transcriptional regulator gene (*W2*; hereafter referred to as *GmPH4*) (Sundaramoorthy et al., 2015). The purple–blue coloration of the flowers of a soybean landrace, Nezumisaya, was identified to be controlled by the *GmPH4* locus (Takahashi et al., 2008). Unlike mutations in soybean structural genes, mutation in the *GmPH4* locus did not change the flavonoid content in the purple–blue flower but increased the pH of petal saps (Iwashina et al., 2008; Takahashi et al., 2008, 2011). Other factors involved in pH determination of the vacuolar sap in soybean have not been determined thus far. In the present study, we characterized two factors that are involved in soybean flower coloration: one is the MYB transcription factor encoded by the regulator gene *GmPH4*, and the other is the putative vacuolar P_3A_-ATPase encoded by *GmPH5*, a new gene identified in this study. Here we have discussed the possible roles of these genes in vacuolar pH regulation of soybean petals.

## Materials and Methods

### Plant Materials

**Figure 1:**
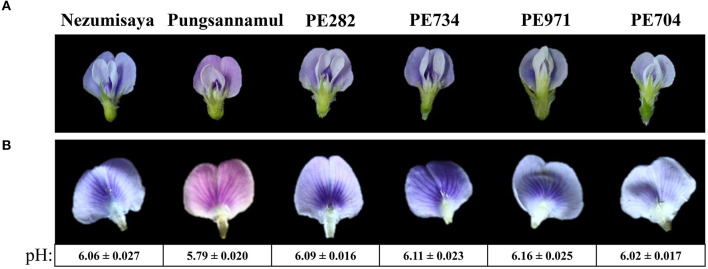
Photographic images showing the flower color of the plant specimens. Images displaying a whole flower **(A)** and a standard petal **(B)** of soybean cultivars Nezumisaya (purple-blue), Pungsannamul (purple), and EMS mutant lines (PE282, PE734, PE971, and PE704; purple-blue). The pH values of the petal homogenates are shown below the standard petals of purple and purple–blue flower lines. Values are presented as mean ± standard error. The result of the *t*-test supports a highly significant difference in pH values between the purple flowers and the purple–blue-flowered lines from WT (*p* < 0.00001).

Four mutant lines (PE704, PE282, PE734, and PE971) with purple–blue flowers (Figure 1A) were isolated from an EMS-induced population of a soybean cultivar [*Glycine max* (L.) Merr], Pungsannamul (Chae et al., 2013). Cultivars such as Pungsannamul, Harosoy, and Jinpung were used in this study. A soybean mutant that previously reported as a purple-blue mutant, Nezumisaya (Iwashina et al., 2008; Takahashi et al., 2008) was used as a mutant control. All the mutant lines were crossed with either Pungsannamul or Jinpung (Table 1), and then segregation analysis was performed. Next, physical mapping was performed using the F_2_ population derived from the crosses of mutant lines PE704 and PE282 with Harosoy, a contrasting cultivar. To conduct the allelism test, breeding crosses were made between mutant lines, and the data are detailed in Table 2. All the experimental populations were grown in the experimental fields at Kyungpook National University (Gunwi, 36°07′N, 128°38′E, Korea).

**Table 1:** Segregation and co-segregation of F_2_ individuals for flower color phenotypes.

**♀**	**♂**	**Total number of plants**	**Phenotype**		**Segre-gation ratio**	**χ^**2**^-value**	***P*-value**	**Genotype**			**Segre-gation ratio**	**χ^**2**^-value**	***P*-value**
			**Purple**	**Purple-blue**				**W^†^**	**H^†^**	**M^†^**			
Pungsannamul	PE704 (*gmph4-p1*)	189	142	47	3:1	0.002	0.96	50	92	47	1:2:1	0.228	0.89
Harosoy	PE704 (*gmph4-p1*)	72	58	14	3:1	1.185	0.27	–	–	–	–	–	–
Pungsannamul	PE282 (*gmph5-a*)	112	85	27	3:1	0.048	0.82	28	57	27	1:2:1	0.054	0.97
Harosoy	PE282 (*gmph5-a*)	154	116	38	3:1	0.009	0.92	–	–	–	–	–	–
Jinpung	PE734 (*gmph5-b*)	137	105	32	3:1	0.197	0.65	37	68	32	1:2:1	0.370	0.83
Pungsannamul	PE971 (*gmph5-c*)	183	136	47	3:1	0.046	0.83	44	92	47	1:2:1	0.104	0.94

† *W, wild homozygote; H, heterozygote; M, mutant homozygote*.

**Table 2:** Allelism analysis of F_2_ individuals for purple-blue flower phenotypes.

**P1 ♀**	**P2 ♂**	**Total number of plants**	**Phenotype**		**Segre-gation ratio**	**χ^**2**^-value**	***P*-value**	**Genotype**			**Segre-gation ratio**	**χ^**2**^-value**	***P*-value**
			**Purple**	**Purple-blue**				**A^†^**	**C^†^**	**B^†^**			
Nezumisaya (*gmph4*)	PE704 (*gmph4-p1*)	95	–	95	–	–	–	–	–	–	–	–	–
Nezumisaya (*gmph4*)	PE282 (*gmph5-a*)	85	47	38	9:7	0.048	0.82	–	–	–	–	–	–
Nezumisaya (*gmph4*)	PE734 (*gmph5-b*)	101	51	50	9:7	0.062	0.92	–	–	–	–	–	–
PE704 (*gmph4-p1*)	PE971 (*gmph5-c*)	71	39	32	9:7	0.057	0.81	–	–	–	–	–	–
PE282 (*gmph5-a*)	PE734 (*gmph5-b*)	71	–	71	–	–	–	21	35	15	–	1.021	0.60
PE282 (*gmph5-a*)	PE971 (*gmph5-c*)	118	–	118	–	–	–	–	–	–	–	–	–

† *A, P1 mutant homozygote; B, P2 mutant homozygote; C, mutant heterozygote*.

### Measurement of pH of the Petal Homogenates

The whole petal limb of an open flower was ground in 1 ml of distilled water, and its pH was measured immediately using a portable pH electrode (Compact pH meter LAQUAtwin-pH-33, Horiba Scientific, Japan). For each line, 10 whole petal limbs were analyzed individually in three replicates. The pH values are presented as mean ± standard error from three independent replications for each line. A comparison between Pungsannamul and the mutant lines was performed using an online *t*-test analysis portal (https://www.usablestats.com/calcs/2samplet). Statistical significance was set as *p* ≤ 0.0001.

### Physical Mapping and Sequence Analysis of *GmPH4* and *GmPH5*

Genomic DNAs were isolated from trifoliate leaves using the cetyltrimethylammonium bromide extraction method (Doyle, 1987). A physical map was constructed from the cross between Harosoy and PE704 using an Affymetrix 180K Axiom® single-nucleotide polymorphism (SNP) array (Affymetrix USA). We selected a total of 14 F_2_ individuals with purple–blue flowers. The region containing the *GmPH4* locus was demarcated by detecting recombinants among the F_2_ individuals using Microsoft Excel. The coding sequences of these candidate genes were amplified using the following PCR conditions: initial denaturation at 94°C for 5 min; 35 cycles of denaturation at 94°C for 20 s, annealing at 55–58°C for 40 s, and extension at 72°C for 1 min, and a final extension at 72°C for 5 min. The PCR products were sequenced (SolGent, Korea) using the sequencing primers listed in Supplementary Table 1.

Another physical map for PE282 was constructed from the cross between Harosoy and PE282. Here, 20 F_2_ individuals with purple–blue flowers were used. The region containing the *GmPH5* locus was demarcated by detecting recombinants among the F_2_ individuals using Microsoft Excel. Fine mapping was performed using specific SNP markers that were developed in this study (data not shown). For next-generation sequencing (NGS) analysis, genomic DNA isolated from Pungsannamul (a wild type) and two mutant lines (PE282 and PE734) were sequenced on an Illumina Hiseq™ 2500 platform (Macrogen, Korea) to construct a paired-end NGS library. The clean reads were rechecked using the FASTQC program for quality control after trimming the adaptor. The clean reads were aligned and mapped to the *G. max* reference genome assembly version 2.0 (Wm82.a2.v1) derived from Phytozome using a Burrows-Wheeler Aligner (BWA) tool with default parameters. The homozygous SNPs between the mutant lines and Pungsannamul cultivar were used for further analysis. The coding sequence of *GmPH5* (*Glyma.03G262600*) was determined using PCR as described in the previous section.

### Multiple Alignment and Phylogenetic Analysis of GmPH5 and P_3A_-ATPase Proteins

P_3A_-ATPase proteins retrieved from the NCBI GenBank (https://www.ncbi.nlm.nih.gov/genbank/) and proteomics databases (https://www.proteomicsdb.org/) were used for multiple alignment analysis that was performed using ClustalW (http://www.genome.jp/tools-bin/clustalw). The P_3A_-ATPase proteins used in the tree construction was derived from the PANTHER classification system for proteins (http://pantherdb.org/) and NCBI database when “GmPH5 protein” was used as a query. The phylogenetic tree was constructed using the MEGA 7.0 software (Kumar et al., 2016).

### Isolation of RNA and qRT-PCR Analysis

Total RNA was isolated from freeze-dried samples using the phenol–chloroform and lithium chloride precipitation methods (McCarty, 1986). The RNA samples were treated with DNase I to remove DNA contaminants (Takara, Japan). First-strand cDNA was synthesized by reverse-transcribing the total RNA with an oligo-dT_(20)_ primer and Superscript III according to the manufacturer's instructions (Invitrogen, USA). Three replicates of relative gene expression quantification was performed according to Park et al. (2015) using the LightCycler® 480 Real-Time PCR System (Roche, Germany). The primers used in this analysis are listed in Supplementary Table 1.

### CAPS and dCAPS Analysis

The genomic DNA isolated from the F_2_ individuals derived from different crosses was used for the cleaved amplified polymorphic sequence (dCAPS) and CAPS analyses. The dCAPS PCR primers (Supplementary Table 1) were designed to detect SNPs in the PE282, PE734, and PE704 mutant lines, together with the Nezumisaya *gmph4* mutant. In Nezumisaya, PE704, and PE282, the nucleotide substitutions (G to A; C to T; and G to A) generated *Hpa*II (CCGG), *Hha*I (GCGC), and *Hha*I (GCGC) sites, respectively, in the PCR products that were amplified from the WT parent. In PE734, the nucleotide substitution (G to A) generated a *Hin*dIII site (AAGCTT) in the PCR product that was amplified from the mutant parent. In PE971, the CAPS PCR primer set was designed to detect the SNP in the mutant line. The nucleotide substitution (C to T) generated a *Bcl*I (TGATCA) site in the PCR products that were amplified from the mutant parent. The PCR conditions were the same as those mentioned in the previous sections. The amplified products were digested and separated on 1.2% agarose gel.

## Results

### Isolation of Soybean Mutants With Purple–Blue Flowers

To identify the genes involved in vacuolar acidification of soybean flowers, we screened an EMS-induced mutant population developed from the Pungsannamul soybean cultivar. We identified four mutant lines (PE704, PE282, PE734, and PE971) with purple–blue flowers (Figure 1A). We measured the pH of petal homogenates of the mutants and the WT cultivar to investigate the physiologic basis of flower color in the mutants (Figure 1B), which may serve as evidence for vacuolar acidification (Faraco et al., 2014). The Pungsannamul cultivar's purple flower had a pH of 5.79, whereas the purple–blue flower of the mutants had a pH value of 6.02–6.16 (Figure 1B). The *t*-test showed that there was a significant difference between the purple and purple-blue flower lines, suggesting that the increased pH in mutant petal homogenates is responsible for the purple–blue coloration. This result is consistent with the increased pH of petal homogenates in petunia blue flower mutants (*ph1*–*ph7*) and Nezumisaya, a soybean purple–blue flower mutant (Iwashina et al., 2008; Takahashi et al., 2013; Faraco et al., 2014).

### Genetic Inheritance Patterns of the New Mutations

The flower colors of the F_2_ individuals derived from the crosses of cultivars (Pungsannamul, Jinpung, and Harosoy) along with those of the mutants (PE282, PE704, PE734, and PE971) were analyzed to investigate the inheritance of mutant alleles in the presence or absence of a purple–blue flower color (Table 1). The segregation patterns of the four F_2_ populations were statistically consistent with a 3:1 ratio (purple:purple–blue). These results indicate that the purple–blue flowers in each mutant are because of a single recessive allele.

### Allelism Test for the *gmph4-p1* and *gmph5* Mutant Alleles

All four mutant lines showed the same phenotype, i.e., purple–blue flower, which led us to perform an allelism test. First, we made crosses between the three mutant lines (PE704, PE282, and PE734) and Nezumisaya, having been previously reported as a *gmph4* mutant line (Takahashi et al., 2008). The resulting flower colors of 95 F_2_ individuals were all identical (purple–blue) in the cross between Nezumisaya and PE704 (Table 2). The result indicated that the purple–blue flower in the PE704 mutant may have been caused by either the same or different alleles of *GmPH4*, hereafter designated as the *gmph4-p1* allele. In contrast, the F_2_ individuals obtained from the populations of PE282 × Nezumisaya and PE734 × Nezumisaya showed a segregation of flower colors to purple and purple–blue in a ratio of 9:7. In addition, we made three more crosses, i.e., PE704 × PE971, PE282 × PE734, and PE282 × PE971. The F_2_ individuals from PE971 × PE704 showed a segregation of flower colors to purple and purple–blue at a ratio of 9:7. Moreover, all F_2_ individuals derived from PE282 × PE734 and PE282 × PE971 crosses showed an identical phenotype, i.e., the purple–blue color. These results suggested that the purple–blue flowers in the PE282, PE734, and PE971 mutants are caused by either the same or different allele of a new gene, which we are to describe as *GmPH5* and hereafter designate as *gmph5-a* in PE282; *gmph5-b* in PE734; and *gmph5-c* in PE971.

### Physical Mapping of the *GmPH4* Locus and Molecular Analysis of the *gmph4-p1* Allele

**Figure 2:**
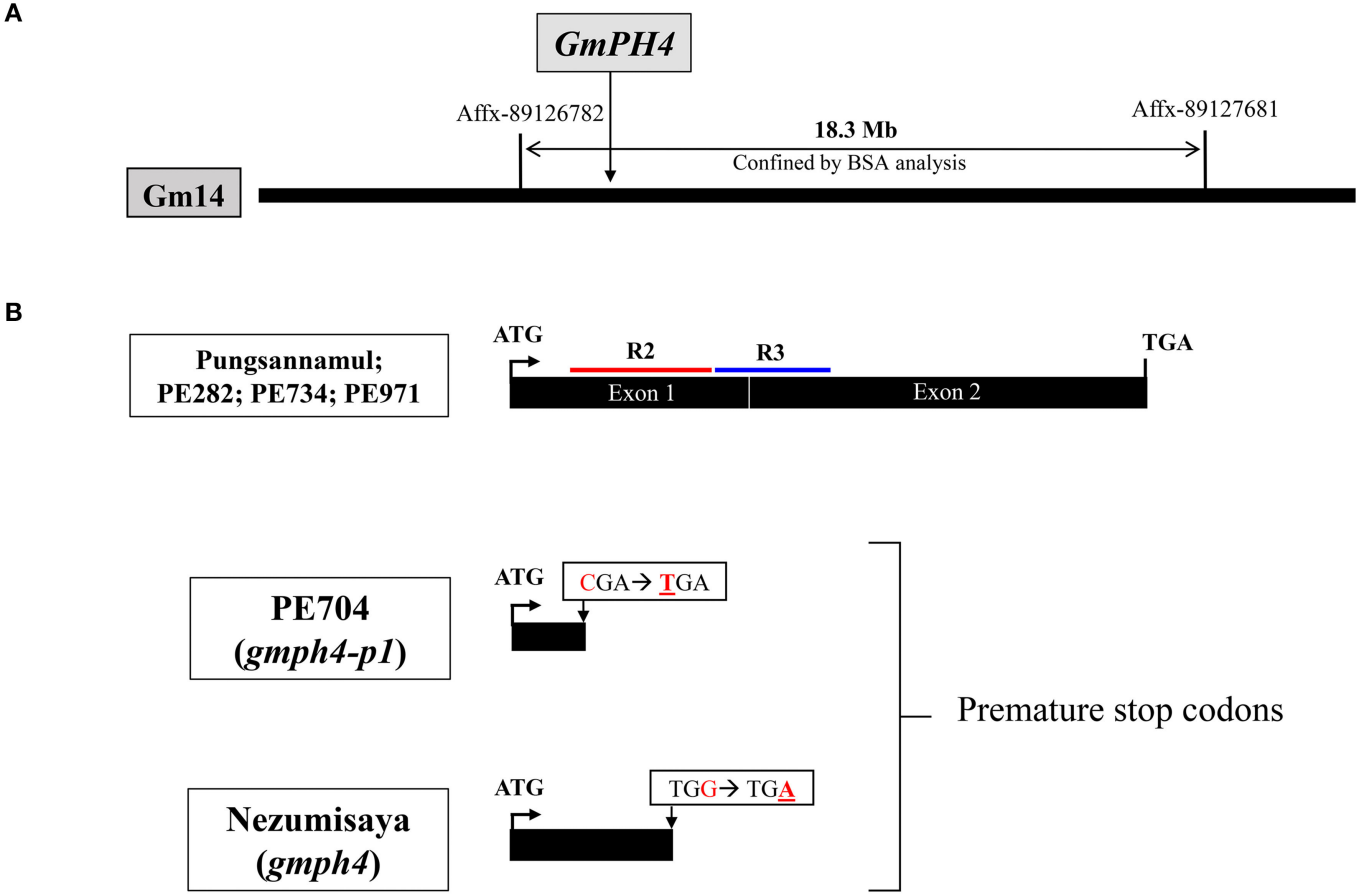
Physical map and gene structure of *GmPH4* in soybean. **(A)** Physical map construction of the *GmPH4* locus. **(B)** Gene structure of *GmPH4* showing the sequence polymorphisms among *GmPH4, gmph4* (Nezumisaya), and *gmph4-p1* (PE704) alleles. Nucleotide substitutions in the mutant alleles are indicated. The regions corresponding to R2 and R3 MYB domains are marked in red and blue, respectively.

We performed physical mapping analysis using an Affymetrix Axiom® SNP array to identify the gene involved in the mutant phenotype of PE704 (*gmph4-p1*). A physical map was constructed using 10 F_2_ individual lines with purple–blue flowers derived from Harosoy × PE704 (Table 1). The locus was mapped to the 18.3 Mb region between Affx-89126782 and Affx-89127681 SNP arrays on chromosome 14 (Figure 2A). In a previous study, the *GmPH4* locus involved in purple–blue soybean flower coloration was genetically mapped in chromosome 14 and was shown to be flanked by the Satt318 marker at a distance of 1.1 cM (Takahashi et al., 2008). Takahashi et al. (2011) previously characterized *GmPH4* (*Glyma.14G154400*) that was located in the region that we mapped. *GmPH4* encodes an MYB transcription factor containing two MYB repeats (R2 and R3) (Figure 2B).

We analyzed the coding sequence of *Glyma.14G154400* (position +1 to 1086) to determine whether *GmPH4* is responsible for the purple–blue flower coloration in the PE704 mutant (Figure 2B). The sequence analysis revealed that PE704 had an SNP (C to T) at nucleotide position 133 of *Glyma.14G154400*. This SNP resulted in a premature stop codon at amino acid position 45. The result indicated that the mutation produced a GmPH4 truncated protein, thereby leading to a complete loss of function, which strongly agrees with the previously reported purple–blue flower mutant line, Nezumisaya, in which its SNP introduced a premature stop codon (TGA) at amino acid position 88 (Takahashi et al., 2011).

### Physical Mapping of the *GmPH5* Locus and Molecular Analysis of the *gmph5* Alleles

**Figure 3:**
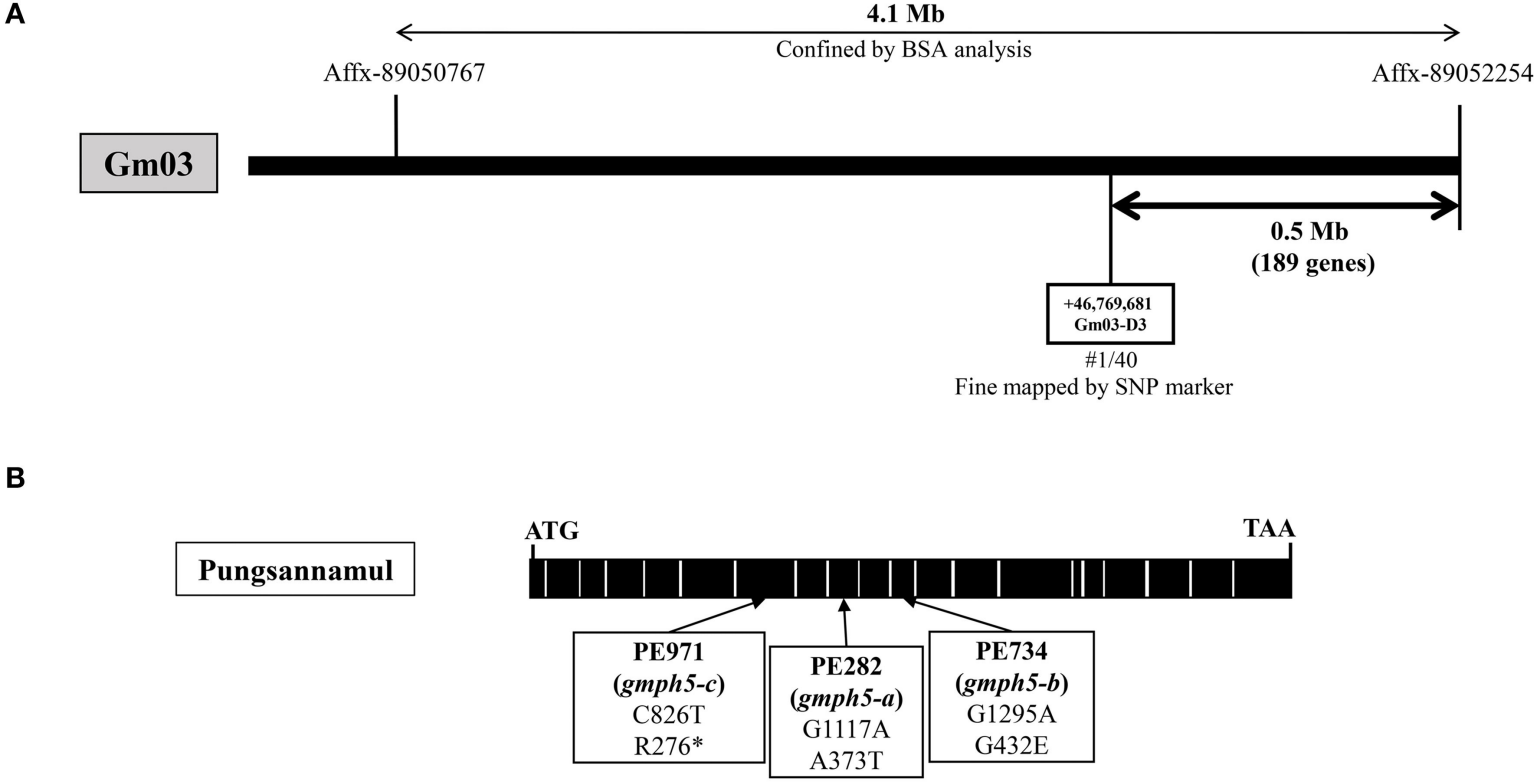
Physical map and gene structure of *GmPH5* in soybean. **(A)** Physical map construction of the *GmPH5* locus. **(B)** Gene structure of *GmPH5* and the sequence polymorphisms between *GmPH5* and *gmph5-a* to *gmph5-c* alleles. Nucleotide substitutions in the mutant alleles are indicated.

To identify the gene responsible for the purple–blue flowers of PE282 (*gmph5-a*), PE734 (*gmph5-b*), and PE971 (*gmph5-c*) mutants, we first used an Affymetrix Axiom® SNP array of 20 F_2_ individuals with purple–blue phenotypes derived from Harosoy × PE282 (Table 1). The *GmPH5* locus was mapped to a 4.1 Mb-spanning region between the Affx-89050767 and Affx-89052254 SNP arrays on chromosome 3 (Figure 3A) in the initial mapping. The mapped region was further narrowed down by the specific SNP markers that were developed in this study. The *GmPH5* locus was mapped to a 0.5 Mb-spanning region between the GM03-D3 marker (Supplementary Table 1) and Affx-89052254.

We performed NGS analysis and obtained data for the PE282 and PE734 mutants to identify the candidate gene. In PE282, each of the five genes in the mapped region had an SNP in their exons, leading to amino acid changes in their respective proteins (Table 3). In PE734, we found that out of the five genes, *Glyma.03G262600* was the only gene with an SNP in the exon, which led to amino acid substitution. We designated it as a candidate gene for *GmPH5* based on the premises that the allelism test indicated that all the mutant alleles (*gmph5-a, gmph5-b*, and *gmph5-c*) corresponded to the same gene, the *Glyma.03G262600* of both *gmph5-a* and *gmph5-b* had SNPs, and *Glyma.03G262600* (position +1 to 2805) was predicted to encode an H^+^ P-ATPase in the Phytozome soybean genome database (https://phytozome.jgi.doe.gov/pz/portal.html#!info?alias=Org_Gmax). The sequence analysis of *Glyma.03G262600* from PE971 (*gmph5-c*) also revealed an SNP (C to T) in the seventh exon and introduced a premature stop codon at the amino acid position 276 (Figure 3B). The SNPs detected in the PE282 (*gmph5-a*) and PE734 (*gmph5-b*) mutant alleles led to amino acid substitutions; the former substituted Thr for Ala (A373T) and the latter substituted Glu for Gly (G432E) as compared with the corresponding sequences of Pungsannamul and Williams 82 cultivars (Table 3, Figure 3B).

**Table 3:** List of candidate genes with point mutations identified in PE282 and PE734 mutant lines.

**Mutant line**	**Gene**	**Annotation**	**Physical position**	**SNP**	**Amino acid change**
PE282 (*gmph5-a*)	*Glyma.03G248400*	Perakine reductase	46420245	G→ A	L→ F
	*Glyma.03G251500*	Serine/threonine-protein phosphatase PP1 isozyme 2-related	46612239	G→ A	L→ F
	*Glyma.03G255700*	Protein of unknown function (DUF1070)	46986507	G→ A	A→ V
	*Glyma.03G256700*	WRKY DNA -binding domain (WRKY)	47074609	G→ A	P→ S
	*Glyma.03G262600*	H^+^ P-ATPase	47535452	G→ A	A→ T
PE734 (*gmph5-b*)	*Glyma.03G262600*	H^+^ P-ATPase	47535630	G→ A	G→ E

**Figure 4:**
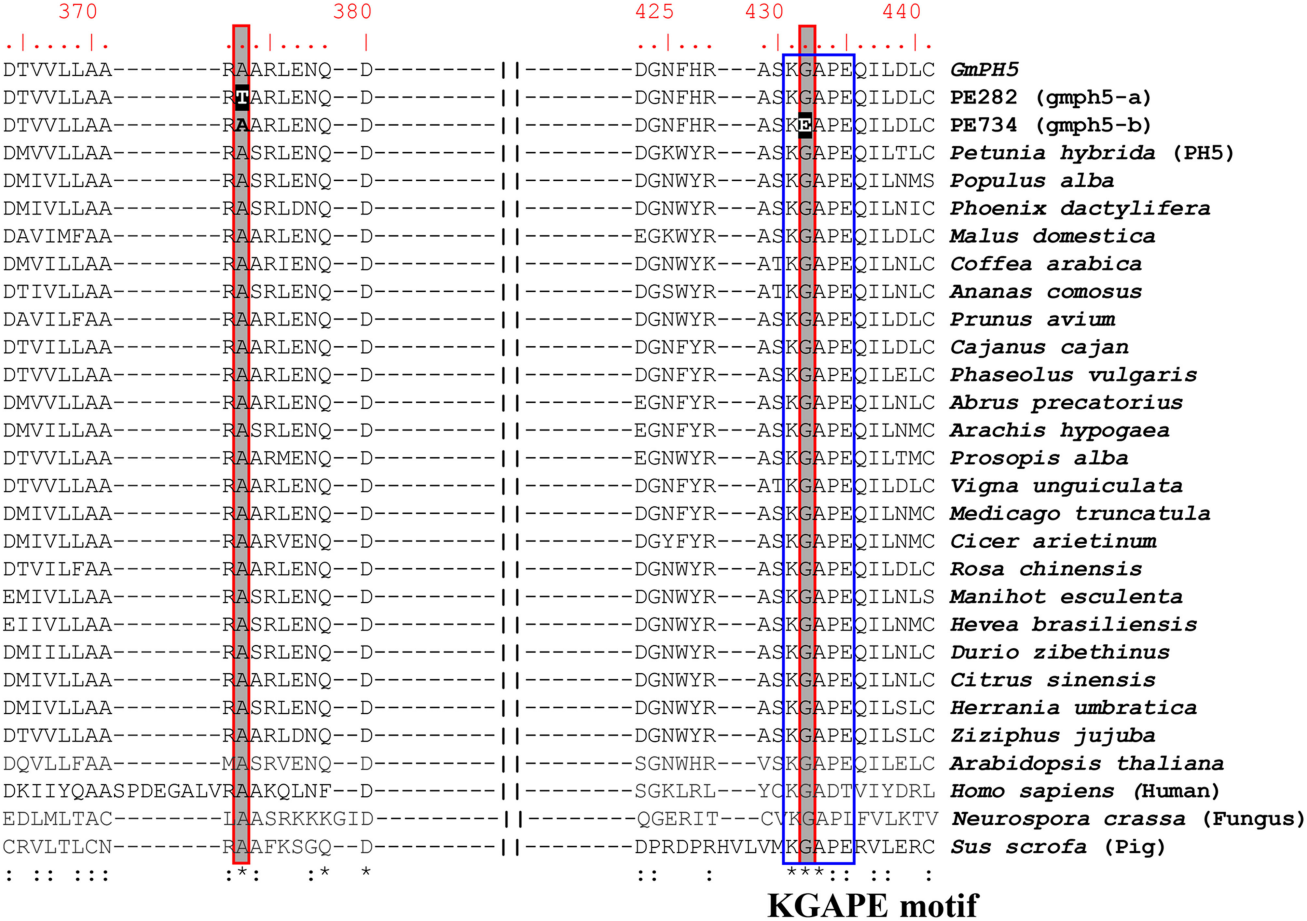
Amino acid sequence alignment of P_3A_-ATPase proteins. Amino acid sequences of P_3A_-ATPase proteins from different species were compared with WT GmPH5 and mutant proteins. SNPs detected in mutants are highlighted in black. The conserved KGAPE motif is highlighted in a blue box. Asterisks indicate identical residues; colons, conserved, or semi-conserved substitutions. GenBank accession numbers of P-ATPase proteins are as follows: *Petunia hybrida*, DQ888719; *Populus alba*, TKR85646; *Phoenix dactylifera*, XP_008783452; *Malus domestica*, XP_008342517; *Coffea arabica*, XP_027124527; *Ananas comosus*, XP_020104345; *Prunus avium*, XP_021819909; *Cajanus cajan*, XP_020211004; *Phaseolus vulgaris*, XP_007139050; *Abrus precatorius*, XP_027348813; *Arachis hypogaea*, RYR52881; *Prosopis alba*, XP_028796327; *Vigna unguiculata*, XP_027902873; *Medicago truncatula*, XP_013450626; *Cicer arietinum*, XP_004494890; *Rosa chinensis*, XP_024181824; *Manihot esculenta*, XP_021610990; *Hevea brasiliensis*, XP_021654241; *Durio zibethinus*, XP_022723470; *Citrus sinensis*, XP_006465725; *Herrania umbratica*, XP_021294688; *Ziziphus jujuba*, XP_015901337; *Arabidopsis thaliana*, 5KSD; *Homo sapiens*, 6K7G; *Neurospora crassa*, 1MHS; and *Sus scrofa*, 5Y0B.

P-ATPases are widely distributed in plants and are involved in transporting diverse small cations and phospholipids (Axelsen and Palmgren, 1998). The P-ATPase transporters have been classified into five major subfamilies (P_1_-P_5_) with subgroups (P_1A−B_, P_2A−D_, P_3A−B_, P_4_, and P_5A−B_) (Sørensen et al., 2019). In the P_3_-ATPase subgroups, P_3A_- and P_3B_-ATPases transport H^+^ and Mg^2+^ ions, respectively. Judging from its homology with petunia PH5 (Verweij et al., 2008; Faraco et al., 2014), *GmPH5* encodes an H^+^ P-ATPase belonging to the P_3A_-type proton pump subfamily. We performed multiple sequence alignment with 26 P_3A_-type ATPases from different plant species along with those from fungus, human, and pig (Figure 4). The resulting alignment showed that the amino acid changes in *gmph5-a* and *gmph5-b* were located at the positions that were highly conserved among all the P_3A_-type ATPases that were analyzed. The PE282 (*gmph5-a*) mutant showed an amino acid change in the catalytically active and cytosolic domain C loop (Novoa-Aponte et al., 2012). The PE734 (*gmph5-b*) mutant showed an amino acid change in the KGAPE motif, which functions in ATP binding (Novoa-Aponte et al., 2012). Based on these findings, we suggest that those SNPs could partially nullify GmPH5 H^+^ P_3A_-ATPase function, which is consistent with the recessive nature of *gmph5-a* and *gmph5-b* as well as the elevated pH values in their petal saps.

A phylogenetic tree was constructed to infer the evolution and relationship of GmPH5 with 46 P_3A_-ATPase proteins of different plant species showing above 80% sequence similarities (Supplementary Figure 1). The phylogenetic tree was split into two major clades; one of which contained species from only three families of angiosperms (Fabaceae, Poaceae, and Brassicaceae) and the other contained the rest of angiosperm species. In both the clades the species were clustered together with the species under their respective families, suggesting that most of the P_3A_-ATPase protein sequences are specific at the family level. The P_3A_-ATPases of all species sharing high homology with the petunia PH5 (Supplementary Figure 1). A previous study (Li et al., 2016) has also revealed the phylogenetic relationship between GmPH5 (GenBank number, XP_003521833) and petunia PH5.

### Co-segregation Analysis Using dCAPS Markers

We developed dCAPS markers for the *gmph4-p1* allele and determined the co-segregation patterns of these markers and the purple–blue phenotypes in PE704 mutant populations (Supplementary Figure 2A). dCAPS analysis showed that the genotype segregation fits a 1:2:1 ratio (Table 1, Supplementary Figure 3A), confirming that the *gmph4-p1* allele is *GmPH4*-recessive. We also performed dCAPS analysis using a *gmph4* marker developed for the Nezumisaya mutant and a *gmph4-p1* marker for the PE704 mutant. Our results showed that the *gmph4* marker was not complemented by the *gmph4-p1* marker, confirming that *gmph4* and *gmph4-p1* are allelic to each other (Table 2).

Because *Glyma.03G262600* was identified as the *GmPH5* gene, we developed dCAPS markers for the *gmph5-a* and *gmph5-b* alleles, as well as a CAPS maker for the *gmph5-c* allele, and determined the co-segregation patterns of the markers and the purple–blue flower color phenotypes (Supplementary Figures 2B–D). All the dCAPS and CAPS markers consistently co-segregated with the flower color phenotypes of F_2_ plants derived from all the segregation crosses (Table 2), confirming that these mutant alleles are strongly associated with the purple–blue flower. The analysis also showed that the genotype segregation fits a 1:2:1 ratio (Table 1, Supplementary Figures 3B–D), confirming that the *gmph5-a, gmph5-b*, and *gmph5-c* alleles are all *GmPH5-*recessive. We also performed dCAPS and CAPS analyses using 71 F_2_ individuals derived from PE282 × PE971. The result showed that the *gmph5-a* marker was not complemented by the *gmph5-c* marker, confirming that *gmph5-a* and *gmph5-c* are allelic to each other.

### Expression Profiles of Genes Involved in Vacuolar Acidification

PH4, AN1, AN11, and PH3 proteins in petunia are MBWW complex components that control the expression of downstream genes, including *PH1* and *PH5*, which are involved in vacuolar acidification (Verweij et al., 2008; Faraco et al., 2014). GmPH4 and GmPH5 showed 60.39 and 83.26% amino acid sequence similarity with petunia PH4 and PH5, respectively (Supplementary Figure 4). With the exceptions of GmPH4 and GmPH5, no other soybean factors that correspond to these petunia proteins have been recently identified, and this prompted us to search for the soybean homologs of PH3, AN1, and AN11 through BLASTP analysis using the “highest densities” criterion, thereby identifying Glyma.19G177400, Glyma.02G147800, and Glyma.06G136900 as GmPH3, GmAN1, and GmAN11, respectively. Their amino acid sequence similarities with the corresponding proteins of petunia were 47.92, 57.86, and 76.70%, respectively (Supplementary Figure 4). As petunia PH1 was reported to enhance PH5 activity (Li et al., 2016), we also identified a soybean homolog of PH1 as GmPH1 (Glyma.05G175300.2) by BLASTP analysis, and the results showed that they shared 67.89% identity (Supplementary Figure 4).

**Figure 5:**
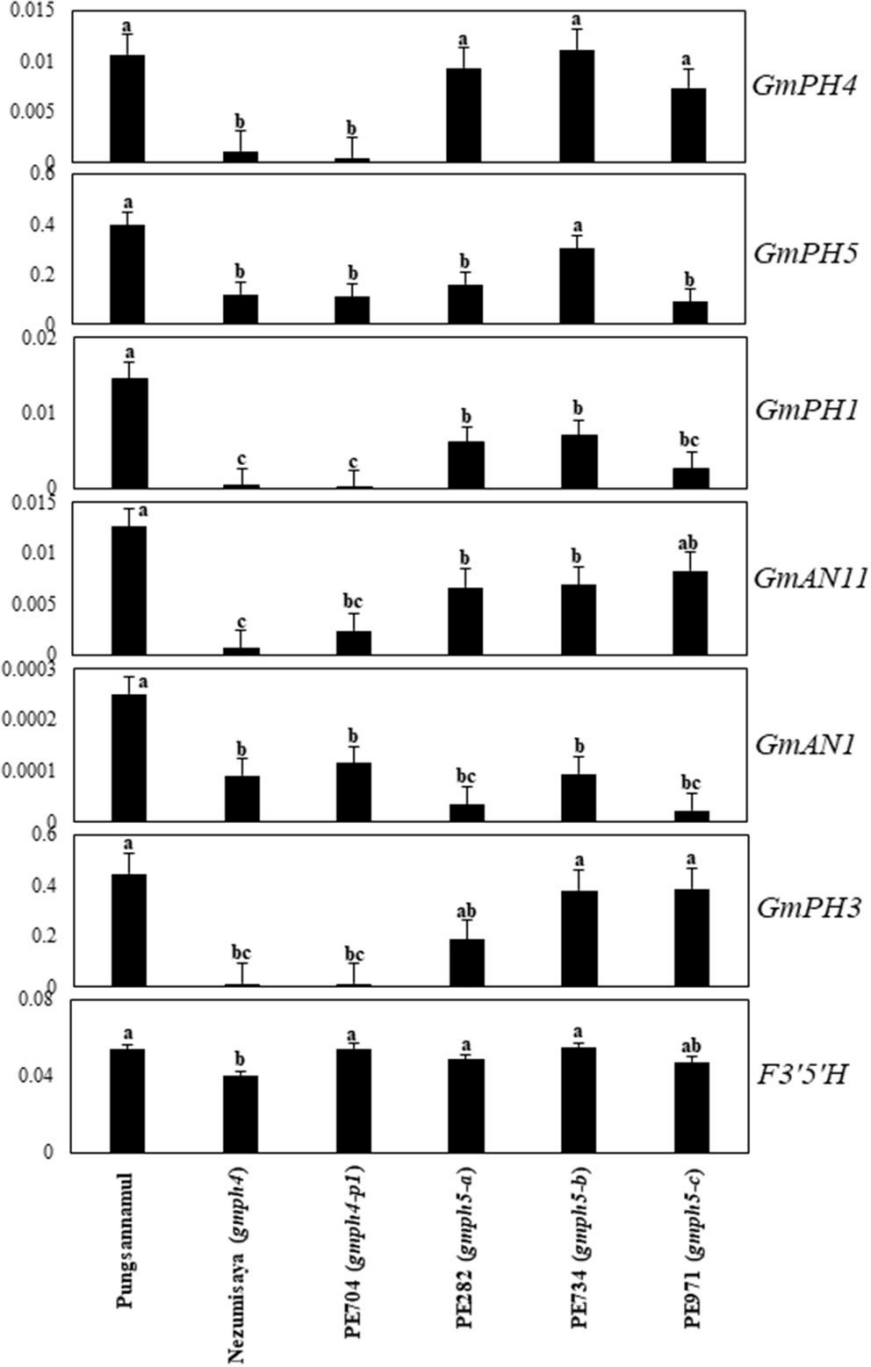
Expression profiles of regulatory and structural genes involved in flower coloration in soybean. qRT-PCR analyses of the regulatory genes (*GmPH4, GmAN11, GmAN1*, and *GmPH3*), and structural genes (*GmPH1* and *GmPH5*) were performed with mRNAs extracted from fully bloomed petals. *F3*′*5*′*H* expression levels are shown for comparison. Error bars represent standard deviation of three biological replicates. The expression of each gene was normalized using constitutive gene 7 (*Cons7*) as the reference gene.

The expression of the aforementioned MBWW complex genes, along with *GmPH1* and *GmPH5*, were analyzed using quantitative real-time (qRT)-PCR analysis using fully bloomed banner petals of the Pungsannamul cultivar and mutant lines (Figure 5). In addition, the *F3*′*5*′*H* (*W1*) gene involved in anthocyanin biosynthesis was analyzed (Sundaramoorthy et al., 2015). PE704 (*gmph4-p1*) and the *gmph4*-mutated Nezumisaya showed a lower level of *GmPH4* transcripts compared with WT Pungsannamul. Interestingly, the transcript levels of *GmPH5, GmPH1, GmPH3, GmAN1*, and *GmAN11* in the *gmph4* mutants were also lower compared with those of the WT. Similar results have been observed in petunia (Faraco et al., 2014). The result indicated that the loss of function of *GmPH4* downregulated all the genes that encode MBWW transcriptional complex components and, consequently, their downstream genes, i.e., *GmPH1* and *GmPH5*. The level of *GmPH1* transcripts was noticeably lower than that of *GmPH5* in both *gmph4* lines. The results are consistent with the findings of Faraco et al. (2014), in which *PH1*, rather than *PH5*, was more prominently influenced by the PH4 MYB transcription factor. All the three *gmph5* mutants showed significant reductions in the transcript levels of *GmPH1* and *GmAN1*, although the reductions in the transcript levels of *GmPH5, GmPH4*, and *GmAN11* were less remarkable. The significant transcript level reductions of the *gmph4-p1* and *gmph5-c* alleles for their respective genes may be caused by the nonsense-mediated mRNA decay to prevent truncated protein expression (Chang et al., 2007; Takahashi et al., 2011). In addition, the low *GmPH5* expression in the *gmph5-a* missense mutant may have been caused by abnormal folding or instability of the GmPH5 protein (Antonarakis and Cooper, 2013). However, neither *gmph4* nor *gmph5* mutations affected *F3*′*5*′*H* expression, which is another gene involved in anthocyanin biosynthesis.

## Discussion

In soybean, the regulatory components involved in vacuolar acidification have remained largely unexplored. In the present study, we explored two of these components to understand the process of vacuolar acidification. Moreover, we isolated four soybean mutants with purple–blue flowers. Among them, one mutant had an allelic *GmPH4* variation that encoded an MYB transcription factor, and its petunia homolog, PH4, was reportedly involved in regulating vacuolar pH (Faraco et al., 2014). The other three mutants had allelic variations in *GmPH5*, which was newly identified as a petunia homolog of *PH5*. Segregation analysis showed that the purple–blue phenotype of *gmph5-a* to *gmph5-c* is controlled by recessive alleles at the *GmPH5* locus. Physical mapping confirmed that *GmPH5* corresponded to *Glyma.03G262600*, which encoded a member of the P_3A_-ATPase family. Finally, the co-segregation study indicated that the *GmPH5* locus is tightly linked to purple–blue flower phenotypes.

The *GmPH4* mutations downregulated *GmPH1* and *GmPH5* expression, which is consistent with the results that showed the increased pH of petal homogenates and the purple–blue hue of petals (Figures 1, 5). This explanation strongly agrees with the petunia system, in which PH1 and PH5 were necessary for vacuolar acidification (Faraco et al., 2014). PH5 proteins can homodimerize to engage in proton-pumping activity and can also form a heterodimeric complex with PH1, thereby enhancing the proton-pumping activity (Faraco et al., 2014). The outcome of PH1–PH5 interaction is involved in vacuolar acidification, thereby modifying flower color (Faraco et al., 2014; Verweij et al., 2016). Notably, *GmPH1* expression was downregulated in the *gmph5* mutant, indicating that the loss of function of the GmPH5 P-ATPase exerted a negative effect on *GmPH1* expression (Figure 5). This phenomenon may be explicable if a feed-forward regulation of GmPH1 by GmPH5 existed.

In petunia, the MBWW (PH4-AN1-AN11-PH3) complex proteins jointly induce *PH1* and *PH5* expression (Faraco et al., 2014). Similarly, the mutation in *GmPH4* downregulated *GmPH1* and *GmPH5* expression, as well as that of *GmPH3, GmAN1*, and *GmAN11*, for the putative components of the MBWW complex in soybean (Figure 5). These results are similar to those of previous studies that evaluated the *ph4* mutant in petunia (Verweij et al., 2008; Faraco et al., 2014). In conclusion, *GmPH5* may encode a new soybean P_3A_-type ATPase gene, whose expression might be regulated by the GmPH4 MYB transcription factor. However, there is a need for further study to prove the interactions between MBWW complex proteins and/or interaction between GmPH1 and GmPH5 in soybean.

## Data Availability Statement

The raw data supporting the conclusions of this article will be made available by the authors, without undue reservation.

## Author Contributions

JS and GTP performed most experiments and wrote the manuscript. J-DL provided the plant materials. GHK, J-DL, and HSS helped in the manuscript writing and discussion. JTS designed the study and supervised all of work. All authors read and approved the final manuscript.

## Conflict of Interest

The authors declare that the research was conducted in the absence of any commercial or financial relationships that could be construed as a potential conflict of interest.
