# Supplementary material for: A P3A-Type ATPase and an R2R3-MYB Transcription Factor Are Involved in Vacuolar Acidification and Flower Coloration in Soybean
Source: Front Plant Sci. 2020 Nov 30;11:580085. doi: 10.3389/fpls.2020.580085 (PMC7793830; doi:10.3389/fpls.2020.580085)
Supplement: Supplementary file 1 [file Data_Sheet_1.PDF]

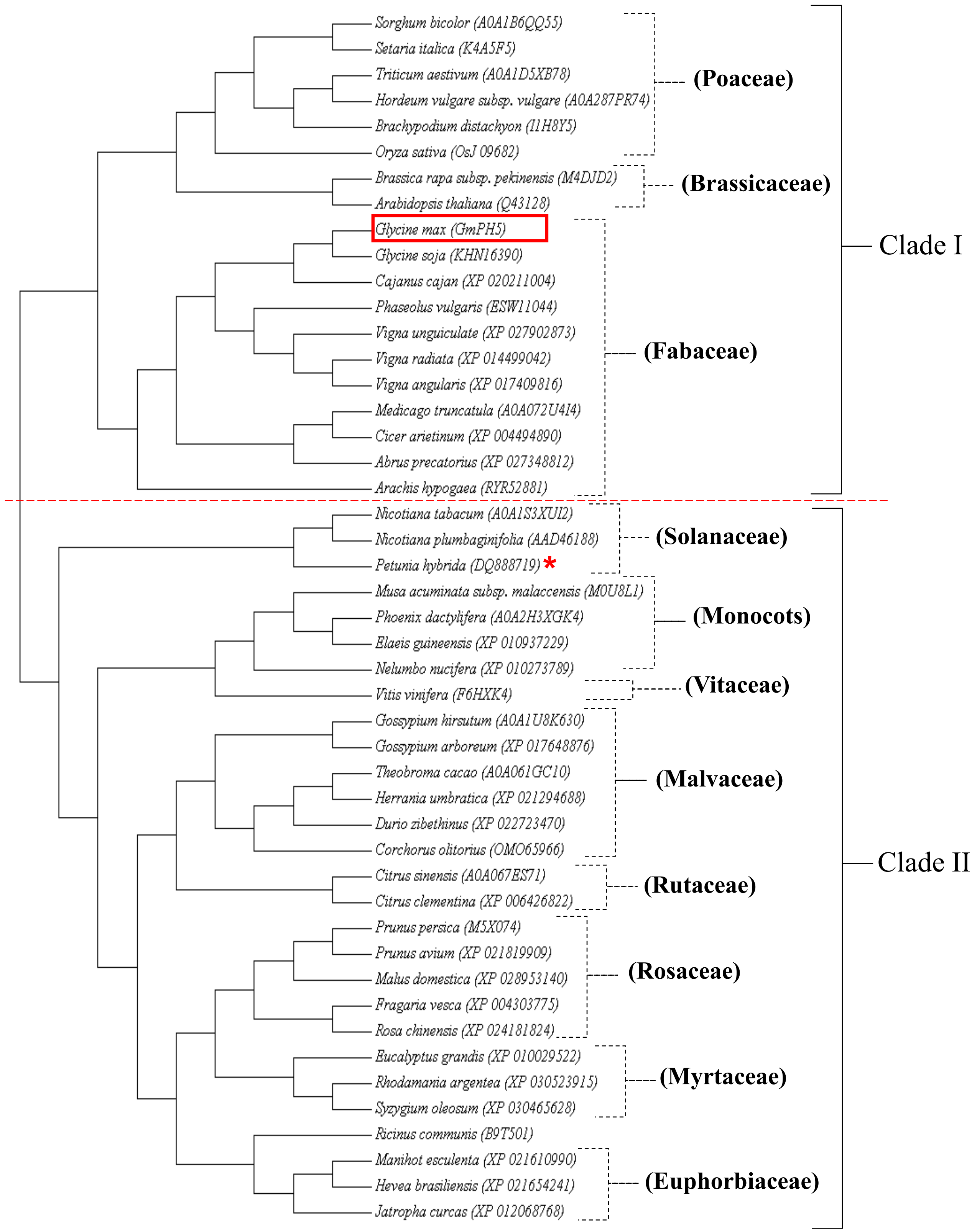

Figure S1. **Cladogram of P_{3A}-ATPase subfamily proteins from different species.** Phylogenetic tree was constructed using 46 P_{3A}-ATPase proteins from distinct species with the highest similarity to GmPH5. The phylogenetic tree was created by the weighted neighbour-joining method using amino acid sequences by MEGA 7.0. The GmPH5 protein was marked by an rectangular red box. The accession number is given in parenthesis for each protein. The accession with an asterisk (*) is petunia PH5.
