# Supplementary material for: A P3A-Type ATPase and an R2R3-MYB Transcription Factor Are Involved in Vacuolar Acidification and Flower Coloration in Soybean
Source: Front Plant Sci. 2020 Nov 30;11:580085. doi: 10.3389/fpls.2020.580085 (PMC7793830; doi:10.3389/fpls.2020.580085)
Supplement: Supplementary file 2 [file Data_Sheet_2.PDF]

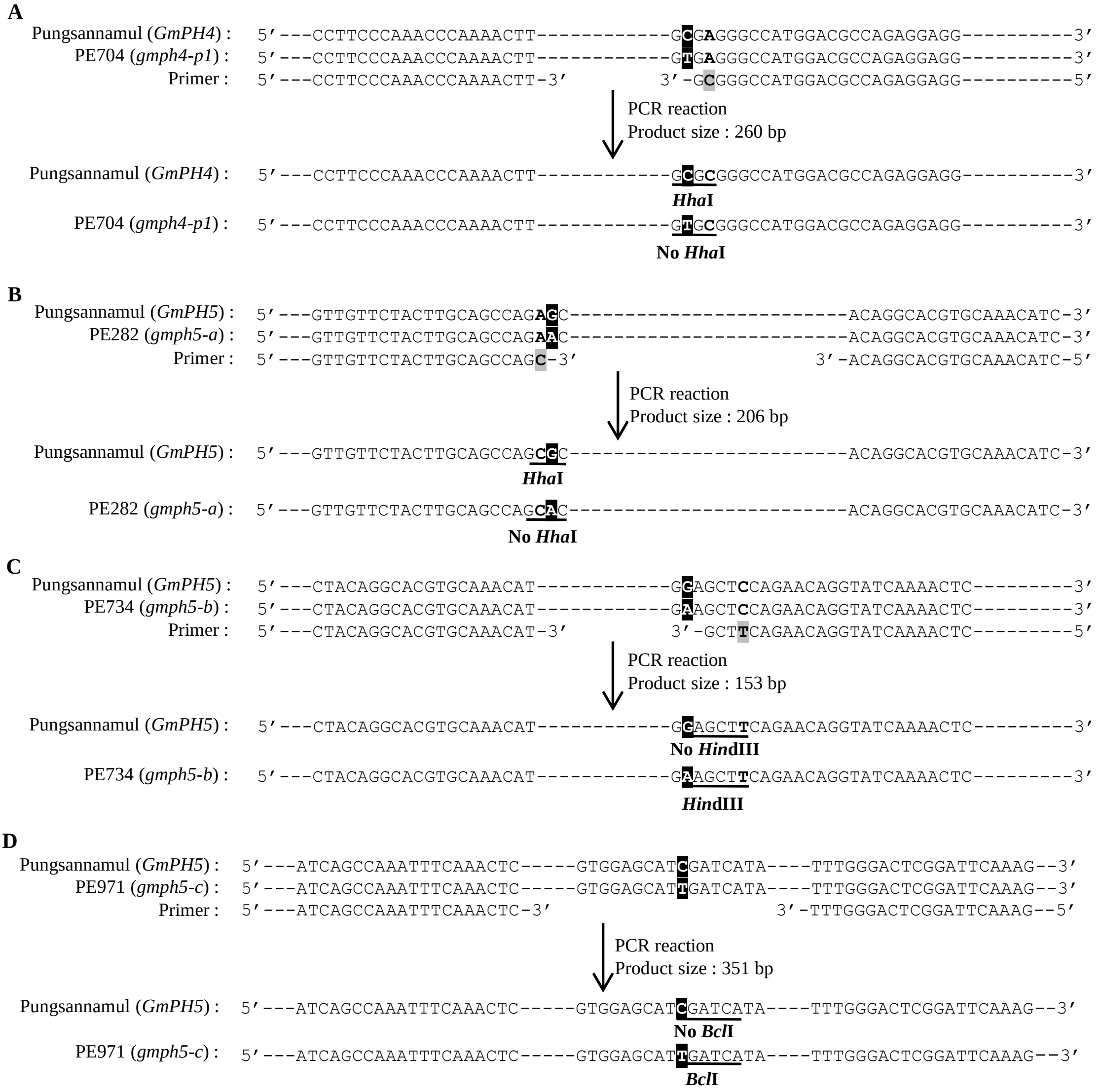

Supplementary Figure S2. Schematic diagram of dCAPS and CAPS analyses. (A) The dCAPS analysis was aimed to detect a point mutation in the *gmph4-p1* allele (highlighted in black). The *Hha*I site (GCGC) underlined was artificially introduced into the PCR products by using a reverse primer with a mismatched base (T, highlighted in gray). The PCR products from Pungsannamul (*GmPH4*) were 260 bp in length and digested with *Hha*I to 220-bp DNA fragments, whereas those from PE704 (*gmph4-p1*) remained uncut after digestion. (B) The dCAPS analysis was aimed to detect a single nucleotide change in the *gmph5-a* allele (highlighted in black). The *Hha*I site underlined was artificially introduced in the PCR products by using a forward primer with a mismatched base (A, highlighted in gray). The PCR products from Pungsannamul (*GmPH5*) were 206 bp in length and digested with *Hha*I to 171-bp DNA fragments, whereas those from PE282 (*gmph5-a*) remained uncut after digestion. (C) The dCAPS analysis was aimed to detect a point mutation in the *gmph5-b* allele (highlighted in black). The *Hin*dIII site (AAGCTT) underlined was artificially introduced into the PCR products by using a reverse primer with a mismatched base (A, highlighted in gray). The PCR products from PE734 (*gmph5-b*) were 153 bp in length and digested with *Hin*dIII to 116-bp DNA fragments, whereas those from Pungsannamul (*GmPH5*) remained uncut after digestion. (D) The CAPS analysis was aimed to detect a SNP in the *gmph5-c* allele (highlighted in black). The *Bcl*I site (TGATCA) underlined was used for the CAPS analysis. The PCR products from PE971 (*gmph5-c*) were 351 bp in length and digested with *Bcl*I into 208- and 143-bp DNA fragments, whereas those from Pungsannamul (*GmPH5*) remained uncut after digestion.
