# Supplementary material for: A P3A-Type ATPase and an R2R3-MYB Transcription Factor Are Involved in Vacuolar Acidification and Flower Coloration in Soybean
Source: Front Plant Sci. 2020 Nov 30;11:580085. doi: 10.3389/fpls.2020.580085 (PMC7793830; doi:10.3389/fpls.2020.580085)
Supplement: Supplementary file 3 [file Data_Sheet_3.PDF]

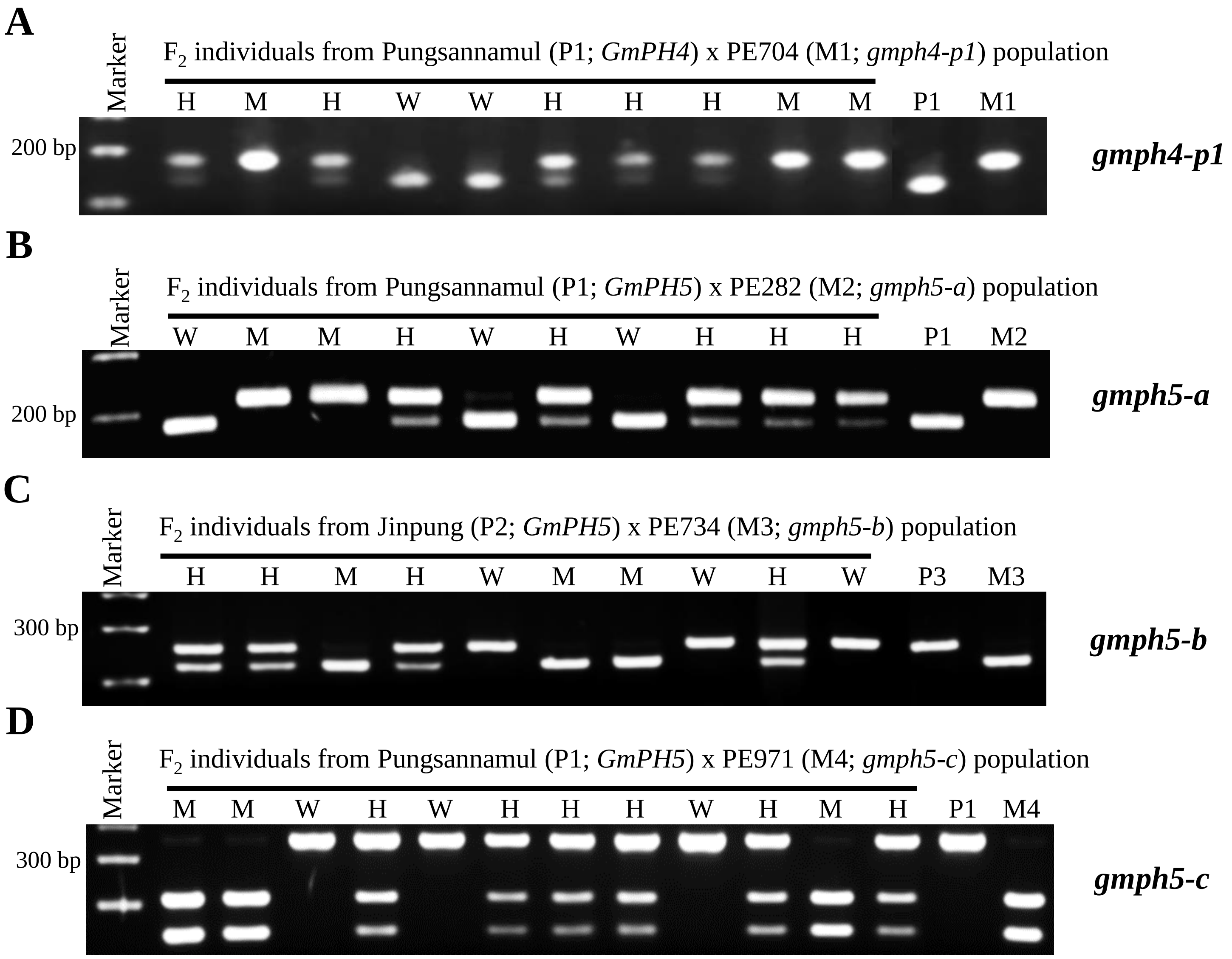

Supplementary Figure S3. Co-segregation analysis of *GmPH4* and *GmPH5* polymorphisms with purple-blue flowers using dCAPS and CAPS markers. (A-D) Co-segregation patterns of *gmph4-p1*, *gmph5-a*, *gmph5-b* and *gmph5-c*. Co-segregation patterns were analyzed in the F2 populations from the segregation crosses listed in Table 1. P1 and P2, the wild cultivars Pungsannamul and Jinpung, respectively (GmPH4 and GmPH5); M1, PE704 (*gmph4-p1*); M2, PE282 (*gmph5-a*); M3, PE734 (*gmph5-b*); M4, PE971 (*gmph5-c*); W, wild-type homozygote; H, heterozygote; M, mutant homozygote.
