# Supplementary material for: A P3A-Type ATPase and an R2R3-MYB Transcription Factor Are Involved in Vacuolar Acidification and Flower Coloration in Soybean
Source: Front Plant Sci. 2020 Nov 30;11:580085. doi: 10.3389/fpls.2020.580085 (PMC7793830; doi:10.3389/fpls.2020.580085)
Supplement: Supplementary file 4 [file Data_Sheet_4.PDF]

Supplementary Figure S4. Sequence alignment of flower color related proteins from petunia and soybean. PH1, PH3, PH4, PH5, PH6 and AN11 proteins from petunia were compared with their respective proteins from soybean. In PH1, GmPH1, PH5 and GmPH5 proteins, the nine conserved motifs of P-Type ATPase family proteins were highlighted in yellow. In PH4 and GmPH4 protein sequence alignment, the R2 and R3 MYB domains are underlined in red and blue, respectively.

PH1 and GmPH1

```
1   ---MWLSNIFPVNHSN----IPYYN-ISQNLVQKPSGQTQHNDGPNTSVFFRFLRRFTSAKKIDGGSRTEEEEKLYSWIYALAQSEKDLVYEYVQSTERG PH1
1   MGRTKV.TL.ANK.N.NNYML..S.T.R.T..NR.N--.HKDRF.FS--L.EL...LIHSR.V..D.K......V...L.T....D.N..F...R..... GmPH1

93  LSFAEADRRLKETGPNIPLENTFPQWWNLLWSASFHPFNIILLVLSVLSYIASDNPNGCIMLILVFISVSLRFYQEFSSSKAAMKLAEFVRCPIKVQRCA PH1
97  ...T......R.N...V...YS..R..H...NSL....I...I......F.TC.S...F..........T......Y.........S...K......... GmPH1

193 GRIVQTEVQVKVDQREVVPGDIVIVGPGDLFPGDVRLLESKHLVVSQSSLTGESATTEKTAYVREDNSTPLLDLKNICFMGTSVVSGSGTGLVVSTGLKT PH1
197 ..V..K.LV.Q....D........FE........I...S..Q.....A......W..D...EI...H..............N....T.....I...SN. GmPH1

293 YLSTIFSKVGKKRPADDFEKGIRHISFVLISIMLVVVSVIVLSVYFTSRDLSKTILYGISVASALTPQMLPLIVNTSLAKGALAMAKDRCIVKSLTAIRN PH1
297 .M..M.......K.P.E....L.R.FYL...VI.A..TIMFVIN.T..LN..QSV.FA.......N.......I..C..................S..H GmPH1

393 MGSMDIICIDKTGTLTVDFATMVNYFDSWGSPNETVLHFAFLNAYFQSQNKHPLDDAIMAYAYTNGFRFQPSKWNKIDEIPFDFTRRRVSVILETKISAK PH1
397 ......L......S..MNH.I...HL.CR.L.Q.KI.RY....S..K.DQ.Y......L.FV.S..........R.........I..........EG--G GmPH1

493 DEKISGNRVLITKGALEDILRICSFVEHIDKGVILTFTKEDYRRISDLAERLSNEGYRVLGLAMKQLLP----EVKVSSMIYEEDVESSMVFVGLISFFD PH1
495 HSQFF.-.F.L.....LEV..V...I.NF..DE.SP.SSN..Q..LN.S.D.....L..IAV.IRK.EMPQIC.TSNG.KRE...I.RD...I...T... GmPH1

589 PPKDSAKQALWRLAEKGVKAKVLTGDTLSLAIRICKEVGIRTTHVITGPDLESLDTDSFHETVKRSTVFARLTPTQKLRVVQSLQTKGDHVVGFLGDGVN PH1
594 .............S...........S...TT.V.R....S........E..Q..QNT.....Q.A..L.....I..Q........I.N........... GmPH1

689 DSLALDAANVGISVDSGASMAKDFANIILLEKDLNVLIAGVEQGRLTFGNTMKYIKMSVIANLGSIISLLIATLIFGFEPLTPMQLLTQNILYNLGQIAI PH1
694 ..........S......VAI...M.D...........V....H...S.......L..........V........L.KY....SR......FI.SV....L GmPH1

789 PWDKMEDCYVKVPQRWSLKGLAMFTLWNGPLCSASDIATLLFLLLYYKVSR-LDFEFFRSAWFVEGLLMQTLIIHLIRTEKIPFIQEVASWPVVCATILI PH1
794 A....DEE...T.HKS.ER..S..M...A.V.TLC.V......WF...AYTDVTQK..H....I....L.................D......IFS.VVT GmPH1

888 SSIGIVIPYTTIGKILGFTALPLSYFGFLVVLFLGYFSFGQIIKKGYILVFKTWL                                             PH1
894 .A...AL.F.P...VM..SLI.........L......TV..VV.RL....Y.K..                                             GmPH1
```

PH3 and GmPH3

```
1   MEVNEAAKIAIARPVASRPRCPIYRSFSELLAGAINTSSTNVHSEMGITAIKPKTVRLKPAANYALIGELSSQVGMSGAPVDCRSDNILQSAEKPKVLYK PH3
1   .HIK..ERVV..K......T.STFK...........A.PAIPS.QTTVS..R.....F...M.QPPS.FVF..ADTF..AL.NS.EMSPKLDT.QSLI.. GmPH3

101 PMAKLAPRKNISLLENKGSYAPDQKREIAEDEAEGHVQSASEVKQQNGLTTESRQSLLAKSRQDKR-IMQSAIVSENTEEVEQSLLNTNNVDRPSYDGYN PH3
101 .T...VSKTTV...A.M.NCSTS--.QQTQQPM.ANF.HSIHE.FRTNTSSNLD..ITPQTEINYQSSEP.KM.QQ.I..DQKV.TSSV.C......... GmPH3

200 WRKYGQKQVKGSEYPRSYYKCTHLKCPVKKKVERSHDGQIAEIVYRGEHNHPKPQPPKRNFSDG-QGRALVSNDTSKETINPALSNQYPHTREANVHRIE PH3
199 .......................PN..........F..N......K.....S...LH...SAA.T..SGV..DG----IVQDMW..SHSERN.G.EV... GmPH3

299 NQADVGLSTQTAYCSKPPCFYDPTSGAGMYRAFRNSED--------SAERDKKLEADCDEPKTKRRKIEGQPNGAGTSGE-SALPHMSIQNTTDSEITED PH3
295 .---T...MHSD.YV.V.RPN.SALNV.ATN.GGV.TENSCGLSGECE.GS.GF..QE...RY....N.N.S.E.AL.E.GLVE.RIVM.SFM....LG. GmPH3

390 GFRWRKYGQKVVKGNSYPRSYYRCTSPKCNVRKFVERTIDDPNALITTYEGKHNHGIPSRRPNSEASKTSSKSSAMKDKS                     PH3
392 ...............P.........NI......H...A....RSFV.........EM.LKNTGTV..ERD.QA.LS...A                     GmPH3
```

PH4 and GmPH4

```
1   MRTP---------------SSSSTTSN-----KVTPCCSKVGLKRGPWTPEEDEILTNYINKEGEGRWRTLPKKAGLLRCGKSCRLRWMNYLRPSVKRGH PH4
1   ..N.SLPTLATTKTKVKNN.NK..NN.NNIGTNYS...NN.............V.A................R........................... GmPH4
                                                                            R2

81  IAPDEEDLILRLHRLLGNRWSLIAGRIPGRTDNEIKNYWNTHLSKKLISHGIDPRTHKPLK--NSNSSSDDITNKLASSSPPSSSKANDLNPILSPTYIS PH4
101 ..............................................NQ..........NPPSIAVP.SST.STIPP.K..PVIIT.NNINPFHHDLTN GmPH4
                                    R3

179 SFQMEEPLGKINTHPGEITSLDDQYQSNAILAEYGDDLNIAVTIEED--------VEMNCCTDDVFSSFLNSLINEDMFACQNQQTNGTFQDFDP----- PH4
201 M.NQGVH.NHLGQ.HQPPPLSNPCDNH.PVP.AAT..VSAMGFMDNNNEDCNNDGINI.YYS.............D.A.EA.Q..HHVQTEIPT.SERSI GmPH4

265 ------------FMASSSTPSSDQ-YNPS--------------------------------                                       PH4
301 NPLPCDDRVDDHHLG.IT.A.AS.G.DDELGVLWESPLVSATFSQHVNDHITKRVHDHHNQ                                       GmPH4
```

PH5 and GmPH5

```
1    MAEDLERPLLGPDNFSREGIDLEKLPLEQVFEELRTSKEGLSDEDAEERLNIFGPNKLEEKRENKFIKFLGFMWNPLSWVMEAAAIMAIALANGGGQGPD PH5
1    ...E.DK...D.E..N.......RI...E...Q....RR...SD...A.IE.........K...IL...S.............L...I......E... GmPH5

101  WQDFVGIVCLLLINSTISFIEENNAGNAAAALMARLAPRTKVLRDGRWQEKDAAILVPGDIISIKLGDIIPADARLLEGDPLKVDQSALTGESLPVTKKT PH5
101  ....I..I...V.........................K.......Q...Q.............................I.............R. GmPH5

201  GDEVFSGSTCKHGEIEAVVIATGVHSFFGKAAHLVDSTQVTGHFQKVLASIGNFCICSIAMGMILEIIVMFPVQNRSYRTGINNLLVLLIGGIPIAMPTV PH5
201  .N............................Y.....E.V.......T..........I...F...I....EH....D................... GmPH5

301  LSVTLAIGSHRLSQQGAITKRMTAIEEMAGMDVLCSDKTGTLTLNRLTIDRNLIEVFQKDMDKDMVVLLAARASRLENQDAIDAAVINMLADPKEARANI PH5
301  ...............................................V........NRN....T........A.........T..V............ GmPH5

401  REVHFLPFNPVDKRTAITYIDSDGKWYRASKGAPEQILTLCQEKQQIAAKVHTIIDKFAERGLRSLAVSFQEIPENSKESPGGPWQFCGLLPLFDPPRHD PH5
401  T..................F..NFH..........D.....D...K..................AY.....K..D......T............. GmPH5

501  SAETIRRALNLGVCVKMITGDQLAIAKETGRRLGMGTNMYPSCSLFGRDKDETEALPVDELIEKADGFAGVFPEHKYEIVKILQMNEHVVGMTGDGVNDA PH5
501  ........................................S..L..E.E.H....I...V.M.......Y...........EKQ............ GmPH5

601  PALKKADIGIAVADATDAARSAADLVLTEPGLSVIVSAVLTSRAIFQRMKNYTIYAVSITIRIVLGFMLLALIWKYDFPPFMVLIIAILNDGTIMTISKD PH5
601  ...........S.....................I..............................A......E........................ GmPH5

701  RVKPSPRPDSWKLNEIFATGVVLGTYLALVTVLFYWLADSTQFFEAHFHVKSLSGSSEEMSSAVYLQVSIISQALIFVTRSQSWSFTERPGALLMFAFVV PH5
701  ......T......P......I.I.............AIVE.T...S....S.I.SD..KV....................RG...L....V...C...I GmPH5

801  AQLVATLIAVYAHISFASVRGIGWGWAGVIWLYSLIFYIPLDIIKFAVCYALTGEAWNLLFDKKTAFTSKKDYGREDREAQWVLSQRSLQRVISPEFEPR PH5
801  ......I.....Y...GKI.....R.........I...V.......T.R.G.S....K.I.ER.....Y.....K.E.A.K----------------.EN GmPH5

901  SRRPSMIAEQAKRRAEITRLRELYTLRGHIESVARLKNLDLNKIQTAHTV                                                   PH5
885  G.GS.L...K.R.....A..G.IHS....VQ..L....F.Q.L..S....                                                   GmPH5
```

PH6 and GmPH6

```
1    ------MQLQTMLRNAVQSVQWTYSLFWQLCPQQGVLVWRDGYYNGAIKTRKTVQPMEVSAEEASLHRSQQLRELYESLSAGESNQPTRRPSAALSPEDL PH6
1    MTAPLDTG..S..QA.....H............VI...G........................Q............V..T.P.....C........ GmPH6

95   TESEWFYLMCVSFSFPAGIGLPGKAYSKKHHIWITGANEVESKVFCRAILAKSARVQTVVCIPLLDGVVELGTTQRIQEDIGFINHVKTFFIEQQPPLPP PH6
101  ...............P.V.......ARRQ.L.L......D..T.S.........I.............F...DKV...LS..Q.......DHLI..R. GmPH6

195  KPALSEHSTSNPTTFSELN--FYSSNTPPSAGTTPADEHGGVAGDEDEEDEDEEDEDEEQEDDEEAELDSDKIAAQVGPADVIAAAEASELMQLDMSEAI PH6
201  ............SSDHIPTVM.TMVD..A.KCNLN.DM-----...I.E.E..E.ED.V.SGS.D.TG-.G..C.T--LRPSTV..P......E.P.D. GmPH6

293  RFGSPDDGSNTNMDSDFHMVGVSQAENPADYQRQAESFKADTSISWAHFQDLPHLPGGPSYDELSQEDTHYSQTVSTILEHLSNQSSKFSSTIMGCISQT PH6
293  .L...N....-.L.....LLA...GG.E.---.....TRRWS.SQEPMQVQ..TSALH.-LED.T...........N..QNQTTR-WLA.PSSI.YNTYS GmPH6

393  TQSAFTRWPSPSTTVSSPFLDGGATSGQWLLKSILFSVPFLHTKYQTAAEVSPKSRDATTVDSSTASRFRKGCSITQEEPSGNHVLAERRRREKLNERFI PH6
387  .H...AK.S.RASHHFH.AA..---TS.....Y...T..H..A.--NPG.S..HTAAD.KLR---------.KGTP.D.L.A................. GmPH6

493  ILRSLVPFVTKMDKASILGDTIEYVKQLRKKVQDLEARANQTEATLQTKDTGTVKVLQGRGKRRMKIVEGSVGGGQAKIT---ASSPSTTHEEEIVQVEV PH6
473  ............................R.I.E....NRL..ERSKLPEVAVQRTSSSSS.EQQRSGVTMMEKRKVR.VEGV.AKAKAVEV.ATTS.Q. GmPH6

590  SIIESDALVELRCPYKEGLLLDVMQMLRELKVEVVTIQSSLNNGSFFAELRAKVKENIYGRKASILEVKKSIHQLIPRV--                    PH6
573  ........L.IE.RHR.............VRI..IGV.......V.V.........HAN.K.V..V...RALN.I..HAVD                    GmPH6
```

AN11 and GmAN11

```
1    MENSSQESQHLRSENSVTYDSTYPIYSMAFSSFPTPRRRIAVGSFIEELNNRVELLSFNEETLTLNPIPNLSFDHPYPPTKLMFHPN--PIKSNNDILAS AN11
1    ....TE..-..........E.P....G.S..-–.SHPH.L.L......Y....DI...HPD..SVT.H.S................RKPSPS.SS.L..T GmAN11

99   SGDYLRLWEVKESSIEPLFTLNNSKTSEYCAPLTSFDWNEVEPKRIGTSSIDTTCTIWDVEKGVVETQLIAHDKEVYDIAWGEAGVFASVSADGSVRIFD AN11
98   .........IRDN.VDAVSLF.......F..........DID.N..A............I.RTL...................R............... GmAN11

199  LRDKEHSTIIYESPTPDTPLLRLAWNKQDLRYMATILMDSNKVVILDIRSPAMPVAELERHQASVNAIAWAPQSCRHICSGGDDGQALIWELPTVAGPNG AN11
198  ..............H...................................TT........RG.........H.ST....A...T.........L.S.T. GmAN11

299  IDPMSMYSAGAEINQLQWSPAQRDWIAIAFSNKLQLLKV                                                              AN11
298  ...VC.....C........A..P.......A..M.....                                                              GmAN11
```
