# Supplementary material for: A P3A-Type ATPase and an R2R3-MYB Transcription Factor Are Involved in Vacuolar Acidification and Flower Coloration in Soybean
Source: Front Plant Sci. 2020 Nov 30;11:580085. doi: 10.3389/fpls.2020.580085 (PMC7793830; doi:10.3389/fpls.2020.580085)
Supplement: Supplementary file 5 [file Data_Sheet_5.PDF]

Supplementary Table S1. List of primers used for PCR.

Analysis	Gene/Physical position	Primer name	Primer sequence (5' → 3')	Restriction enzyme
SNP marker	GM03-D3 (+46769681)	GM03-dcaps-D3-F	CCAAGAGGGGCGCGATTTTACCTATTCAGCTGCCATTTCTA**G**	HhaI
		GM03-dcaps-D3-R	ACAATCTTACATCCAACTTAG	
Genomic sequence (coding region)	*Glyma.14G154400* (*GmPH4*)	GmPH4-F1	AAATGATTGAATTTTATTCTGAATCTC	–
		GmPH4-R1	ACCTTGGTCTTGGTTGTTGC	
		GmPH4-F2	CCTTCCCAAACCCAAAACTT	–
		GmPH4-R2	TGGAGGAGGTTTAGAAGGTGGA	
		GmPH4-F3	CAAGAACCCACAAGCCTCTT	–
		GmPH4-R3	TGAAGCTGGTAACCAATAATAACAA	
	Glyma.03G262600 (*GmPH5*)	GmPH5-F1	GCCTTCGGTCATTGTTTGTT	–
		GmPH5-R1	GGTTGTAACTCGGTGAACAGG	
		GmPH5-F2	CCCTGTTCACCGAGTTACAA	–
		GmPH5-R2	CCTGAAAAGACCTCGTTACCA	
		GmPH5-F3	CAATCGATCAAGAATGGAGATG	–
		GmPH5-R3	AGCACTCAGCAGCACAAAGA	
		GmPH5-F4	CGCCCGCATACTTTATATCG	–
		GmPH5-R4	TCAACATTGTGGCAGGTAGC	
		GmPH5-F5	TCATTATGTTCCCCTTCTTGC	–
		GmPH5-R5	GGCAAGCAATGGGTAATTTG	
		GmPH5-F6	GCAAATTACCCATTGCTTGC	–
		GmPH5-R6	GCAATGATCAGGACCATGAA	
		GmPH5-F7	GTCAAACCAAACCCTTTTGC	–
		GmPH5-R7	CATCAGTAGAACCCCTGGTCTC	
		GmPH5-F8	TTTATGCAGAGCCACTTCCA	–
		GmPH5-R8	CCGAGAGCCATATTCTGCAT	
qRT-PCR	*GmPH4*	GmPH4-qRT-F	CTGGGCAATCGGTGGTCTTT	–
		GmPH4-qRT-R	AGGAACGGCAATAGATGGTG	
	GmPH5	GmPH5-qRT-F	AGTTGGAAGCTGCCTGAGAT	–
		GmPH5-qRT-R	ACTTTCTCGCTGTCACTGGA	
	GmPH1	GmPH1-qRT-F	CAGGCTTAGTGATTTCCACA	–
		GmPH1-qRT-R	AAACATGATGGTAACAACTG	
	F3'5'H	F3'5'H-qRT-F	GAAAGGCACTTGATGATTGG	–
		F3'5'H-qRT-R	TCAAACACTCGACGACTCAA	
	GmAN11	GmAN11-qRT-F	GGAAAACTCCGTCACTTACGAG	–
		GmAN11-qRT-R	AGGGTGGAAAGAGAGGATGTCG	
	GmPH6	GmPH6-qRT-F	GCAGCTGGAGATGCCAGAGGACATT	–
		GmPH6-qRT-R	TTCTTGACTTGAGCTCCACCTT	
	GmPH3	GmPH3-qRT-F	AACCAACACGAGCTCAAATCTT	–
		GmPH3-qRT-R	ACCATCATAAGAAGGTCGATCACAATTG	
	DFR2	DFR2-qRT-F	GGAACACCCAAACCCTGTCA	–
		DFR2-qRT-R	GGCATAAGAAAGGGGCCAAC	
dCAPS	*gmph4* (Nezumisaya)	dCAPS-gmph4-Nez-F	CGAAGCGCGCCGGCCTTCTCCGCTGCGGCAAGAGCTGCCGCCTCCGC**C**G	HpaII
		dCAPS-gmph4-Nez-R	TCTTGGATCAATCCCTTGGT	
	gmph4-p1 (PE704)	dCAPS-gmph4-p1-F	CCTTCCCAAACCCAAAACTT	HhaI
		dCAPS-gmph4-p1-R	TTCGCTAACACCTCGTCCTCCTCTGGCGTCCATGGCCC**G**C	
	gmph5-a (PE282)	dCAPS-gmph5-a-F	GGACAAGGATACGGTTGTTCTACTTGCAGCCAG**C**	HhaI
		dCAPS-gmph5-a-R	GATGTTTGCACGTGCCTGTA	
	gmph5-b (PE734)	dCAPS-gmph5-b-F	CTACAGGCACGTGCAAACAT	HindIII
		dCAPS-gmph5-b-R	ATCTTTCATGAGTTTTGATACCTGTTCTG**A**AGCT	
CAPS	*gmph5-c* (PE971)	CAPS-gmph5-c-F	CAATCAGCCAAATTTCAAACTC	BclI
		CAPS-gmph5-c-R	CTTTGAATCCGAGTCCCAAA	
